# The significance of serum sST2 and cfDNA in children with severe pneumonia complicated by myocardial damage

**DOI:** 10.5937/jomb0-51197

**Published:** 2025-03-21

**Authors:** Tingting Zhao, Ye Liu, Haoran Jia, Dexing Wang, Meng Du, Weiwei Wang

**Affiliations:** 1 Baoding Hospital of Beijing Children's Hospital Affiliated to Capital Medical University, Neonatology, Baoding, China; 2 Baoding Hospital of Beijing Children's Hospital Affiliated to Capital Medical University, Pediatrics, Baoding, China

**Keywords:** sST2, cfDNA, severe pneumonia complicated by myocardial damage, cardiac functions, sST2, cfDNA, teška upala pluća, komplikovana oštećenjem miokarda, srčane funkcije

## Abstract

**Background:**

The paper aimed to explore the significance of serum soluble ST2 (sST2) and circulating cell-free DNA (cfDNA) in predicting cardiac functions in children with severe pneumonia complicated by myocardial damage.

**Methods:**

This case series study evaluated the serum sST2 and cfDNA levels of 60 children with severe pneumonia complicated by myocardial damage, assessing clinical data, biomarker levels, and cardiac function.

**Results:**

We analyzed data from a cohort of 60 patients with a mean age of 4.47±1.88 years and a male: female ratio of 28:32. At baseline, patients had elevated levels of serum biomarkers, including sST2 and cfDNA, which were associated with cardiac function parameters and clinical outcomes. After 6 months, patients showed significant correlations between sST2, cfDNA, and cardiac function parameters, including left ventricular end-diastolic diameter (LVEDd), left ventricular end-systolic diameter (LVESd), and E/A ratio. Multivariate analysis revealed that higher levels of sST2 and cfDNA were associated with increased LVEDd, LVESd, and E/A ratio, as well as a lower likelihood of improvement and a higher likelihood of 6-month readmission.

**Conclusions:**

These findings suggest that sST2 and cfDNA may be useful biomarkers for predicting cardiac function and outcomes in this patient population.

## Abstract (sr)

**Uvod:**

Rad je imao za cilj da istraži značaj serumskog rastvorljivog ST2 (sST2) i slobodne cirkulišuće DNK (cfDNA) u predviđanju srčanih funkcija kod dece sa teškom upalom pluća, komplikovanom oštećenjem miokarda.

**Metode:**

Ova studija serije slučajeva je izvršila evaluaciju nivoa serumskog sST2 i cfDNK kod šezdesetoro dece sa teškom upalom pluća komplikovanom oštećenjem miokarda, analizirajući kliničke podatke, nivoe biomarkera i srčane funkcije.

**Rezultati:**

Analizirali smo podatke iz grupe od 60 pacijenata sa prosečnom starošću od 4,47±1,88 godina i odnosom muško:ženski - 28:32. Na početku studije, pacijenti su imali povišene nivoe serumskih biomarkera, uključujući sST2 i cfDNA, koji su bili povezani sa parametrima srčane funkcije i kliničkim ishodima. Nakon šest meseci, kod pacijenata su uočene značajne korelacije između sST2, cfDNA i parametara srčane funkcije, uključuju}i krajnji dijastolni prečnik leve komore (LVEDd), krajnji sistolni prečnik leve komore (LVESd) i E/A racio. Multivarijantna analiza je pokazala da su viši nivoi sST2 i cfDNA bili povezani sa povećanim LVEDd, LVESd i raciom E/A, kao i sa manjom verovatnoćom poboljšanja stanja i većom verovatno}om ponovnog prijema u bolnicu u roku od šest meseci.

**Zaključak:**

Ovi rezultati ukazuju da sST2 i cfDNA mogu da budu korisni biomarkeri za predviđanje srčane funkcije i ishoda u ovoj populaciji pacijenata.

## Introduction

Severe pneumonia is a common serious respiratory disease in paediatrics, and one of its complications is myocardial damage, which not only increases the mortality of children but also affects their long-term health [1] [2]. Therefore, effective prevention and treatment of severe pneumonia complicated with myocardial damage is of great significance in advancing children’s therapeutic effect and mass of life [3] [4]. It also causes a series of diseases like arrhythmia, increasing the risk of heart failure and sudden death in children [5]. Therefore, monitoring and preventing cardiac function decrease in children with severe pneumonia is of great clinical value. Serum sST2 and cfDNA, as emerging biomarkers in recent years, have shown important potential applications in the diagnosis and prognosis evaluation of heart diseases [6]. SST2 is a marker related to cardiac stress and inflammatory reaction, and the increase in its level is closely related to the increased risk of myocardial injury and heart failure [7]. CfDNA is a free DNA fragment released into the blood by apoptosis or necrosis, and its level in myocardial injury is also of great significance [8] [9]. Severe pneumonia complicated with myocardial damage is a serious clinical situation, and when it happens, the prognosis of patients is often more unfavourable [10] [11] [12]. In the context of severe pneumonia, the atrium may be damaged by an inflammatory reaction, which can be detected by changes in the electrocardiogram, increased cardiac biomarkers, or changes in cardiac imaging technology (such as echocardiography) [13] [14]. Severe pneumonia affects the lungs and causes a systemic inflammatory reaction, which affects the heart function and then leads to myocardial damage [15]. In this case, the level changes of sST2 and cfDNA can provide important information for the clinic. SST2 is a biomarker closely related to cardiac stress and inflammation, which rises during myocardial stress and can reflect myocardial cells’ stress state and degree of inflammation. The increase in sST2 level is closely related to the prognosis of heart diseases, so monitoring sST2 level in children with severe pneumonia complicated with myocardial damage can help evaluate cardiac involvement’s degree and prognosis [16] [17]. The combined monitoring of these two markers provides a new perspective and method for assessing myocardial damage and cardiac functions. Against this background, this study aimed to explore the influence of serum sST2 and cfDNA on predicting cardiac functions in children with severe pneumonia complicated with myocardial damage.

## Materials and methods

This was a case series study from April 2021 to December 2023 in which 60 children with severe pneumonia complicated with myocardial damage were recruited as the research object before being included in the study. The patient or their guardian informed consent and signed the study consent form.

Entry criteria were as follows: participants are between 1 and 15 years old; a diagnosis of severe pneumonia confirmed by a doctor; cardiac ultrasound or other related examination confirmed the existence of myocardial damage. We obtained the written informed consent of parents or legal guardians. There were no other major acute or chronic medical conditions except severe pneumonia and myocardial damage. Parents or guardians agreed and could make children participate in long-term follow-up.

Exclusion criteria were: known chronic heart disease or myocarditis diagnosed before; at the same time, there were other serious systemic diseases, such as advanced nephropathy, severe liver disease or malignant tumour; recently (within 3 months) received treatment from other clinical trials; unable to obtain reliable blood samples or imaging data.

Our hospital’s Ethics Committee approved this study. The patient or their guardian was informed of the research content and was willing to cooperate with the doctor.

### Outcomes and measures

ELISA was used to test the serum sST2 and cfDNA levels and the markers of troponin, IL-6 and TNF-α. These data helped to evaluate the inflammatory state and its changes after interventions. Cardiac function parameters such as LVEDd, LVESd and E/A ratio were collected by echocardiography to assess patients’ cardiac function changes after treatments. In addition, this study also compared the improvement rate of cardiac function, the remission rate of cardiac functions and the hospitalization rate within 6 months.

### Statistical analysis

In this study, all the data were processed by SPSS20.0 statistical analysis software (IBM); the measurement data were measured by »mean ± standard deviation« (± s), one-way analysis of variance or repeated measures, LSD-t-test, the count data were calculated by percentage (%), and χ^2^; P<0.05 represents statistical significance.

## Results

**Figure 1:**
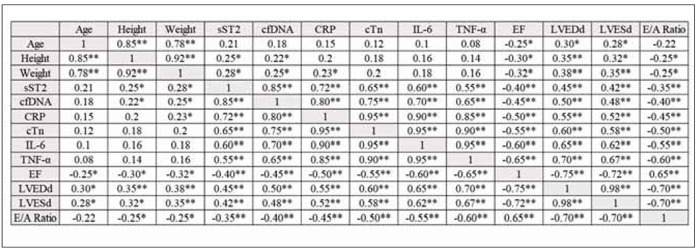
Correlation matrix of study variables (r values of Pearson correlation is shown).<br>**: p < 0.01, *: p < 0.05

Our cohort of patients had male:female ratio of 28:32, the mean age was 4.47±1.88 years, the mean height was 118.49±3.52 cm, and the average weight was 24.46±2.69 kg, among which 3 patients had a family history of heart disease, and 1 case had previous heart and lung history (Table 1). Figure 1

**Table 1:** Detailed information on single nucleotide polymorphisms (SNPs) related to leakage factors and outcome factors.

Category	Unit	Baseline	6 month follow up
Gender	Male:<br>Female	1.188889	
Age	years	4.47±1.88	
Height	cm	118.49±3.52	
Weight	kg	24.46±2.69	
Family History of Heart Disease	%	3 (5.00%)	
Past Medical History of Heart and Lung	%	1 (1.66%)	
sST2	ng/mL	30.46±3.21	28.71±2.65
cfDNA	ng/mL	11.45±1.37	10.33±1.20
CRP	mg/L	21.45±1.63	18.61±1.44
cTn	ng/mL	0.06±0.01	0.05±0.02
IL-6	pg/mL	26.38±2.59	23.28±2.16
TNF-α	pg/mL	28.57±3.11	24.31±2.55
EF	%		55.49±3.51
LVEDd	mm		40.63±2.88
LVESd	mm		29.56±2.35
E/A Ratio	ratio		1.27±0.10
Improvement Rate of Cardiac Function	%		0.5
Remission Rate of Cardiac Functions	%		0.45
6-month Readmission Rate	%		0.2833

The correlation matrix shows several significant relationships between the variables. Age, Height, and Weight are highly correlated with each other (r>0.85), indicating that they tend to increase together. sST2, cfDNA, CRP, cTn, IL-6, and TNF-α are highly correlated (r>0.60), suggesting that they tend to increase together. These variables are all related to inflammation and cardiac function. EF is negatively correlated with LVEDd, LVESd, and E/A Ratio (r<-0.65), indicating that these variables tend to decrease as EF increases. Table 2

**Table 2:** Multiple Linear Regression Analysis of Serum sST2 and cfDNA on Cardiac Function Parameters at 6-Month Follow-up and Logistic Regression of Serum sST2 and cfDNA on Improvement and Readmission Rates. **Significant at p<0.05

	LVEDd (mm)	LVESd (mm)	E/A Ratio	Improvement Rate	6-month Readmission
	β (95% CI)	β (95% CI)	β (95% CI)	Odds Ratio (95% CI)	Odds Ratio (95% CI)
Serum sST2 (ng/mL)	0.43 (0.21–0.65)*	0.31 (0.12–0.50)*	0.21 (0.08–0.34)*	1.23 (1.05–1.44)*	1.50 (1.20–1.88)*
cfDNA (ng/mL)	0.35 (0.15–0.55)*	0.26 (0.09–0.43)*	0.18 (0.05–0.31)*	1.18 (1.02–1.36)*	1.38 (1.12–1.70)

We found significant associations between serum biomarkers, echocardiographic parameters, and clinical outcomes. For every 1 ng/mL increase in serum soluble ST2 (sST2), there were significant increases in left ventricular end-diastolic diameter (LVEDd, β=0.43, 95% CI: 0.21–0.65, p<0.05), left ventricular end-systolic diameter (LVESd, β=0.31, 95% CI: 0.12–0.50, p<0.05), and E/A ratio (β=0.21, 95% CI: 0.08–0.34, p<0.05). Similarly, every 1 ng/mL increase in circulating cell-free DNA (cfDNA) was associated with significant increases in LVEDd (β=0.35, 95% CI: 0.15–0.55, p<0.05), LVESd (β=0.26, 95% CI: 0.09–0.43, p<0.05), and E/A ratio (β=0.18, 95% CI: 0.05–0.31, p<0.05). Furthermore, higher levels of sST2 and cfDNA were associated with a lower likelihood of improvement (sST2: odds ratio = 1.23, 95% CI: 1.05-1.44, p<0.05; cfDNA: odds ratio = 1.18, 95% CI: 1.02–1.36, p<0.05) and a higher likelihood of 6-month readmission (sST2: odds ratio = 1.50, 95% CI: 1.20–1.88, p<0.05; cfDNA: odds ratio = 1.38, 95% CI: 1.12–1.70, p<0.05).

## Discussion

Our results showed that higher levels of sST2 and cfDNA were associated with increased left ventricular end-diastolic diameter (LVEDd), left ventricular end-systolic diameter (LVESd), and E/A ratio, as well as a lower likelihood of improvement and a higher likelihood of 6-month readmission.

Our results align with previous studies demonstrating the prognostic value of sST2 in pediatric cardiac diseases. For instance, elevated sST2 levels have been associated with an increased risk of adverse events in pediatric dilated cardiomyopathy (PDCM) [17] Similarly, sST2 is a useful biomarker for predicting cardiac outcomes in adult patients with complex congenital heart disease [17]. Our study extends these findings by demonstrating the potential of sST2 as a biomarker in children with severe pneumonia complicated by myocardial damage.

The analytical performances of sST2 assays have been previously evaluated, and reference intervals have been established for children and adolescents [18]. Our study highlights the clinical application of sST2 as a biomarker, demonstrating its potential in predicting cardiac function and outcomes in pediatric patients.

The mechanisms underlying the association between sST2 and cardiac outcomes are not fully understood. However, it is thought that sST2 may reflect the degree of cardiac stress and inflammation, which are common in pediatric cardiac diseases [19]. Further studies are needed to fully elucidate the mechanisms underlying the association between sST2 and cardiac outcomes.

The use of cfDNA as a biomarker in pediatric cardiac diseases is a growing area of research. A recent Tanem et al. [20] study demonstrated that nuclear cell-free DNA (ncfDNA) kinetics can predict adverse events after pediatric cardiothoracic surgery. The study found elevated preoperative ncfDNA was strongly associated with postoperative arrest and extracorporeal membrane oxygenation. Our study builds on this research by investigating the role of cfDNA in predicting cardiac functions in children with severe pneumonia complicated by myocardial damage. Another study by Richmond et al. [21] validated donor fraction cell-free DNA as a noninvasive test to assess the risk of acute cellular rejection and antibody-mediated rejection after heart transplantation in pediatric and adult recipients. The study found that donor fraction cell-free DNA at a threshold of 0.14% had excellent negative predictive value for detecting rejection. While our study focused on the use of cfDNA in predicting cardiac functions, these studies collectively highlight the potential of cfDNA as a biomarker in pediatric cardiac diseases.

## Dodatak

### Funding

Baoding Hospital of Beijing Children’s Hospital Affiliated to Capital Medical University, supports the research: The value of serum sST2 and cfDNA in predicting myocardial damage in children with severe pneumonia (No. 2341ZF390).

### Conflict of interest statement

All the authors declare that they have no conflict of interest in this work.
